# Cholesterol-Bearing Fluorescent G-Quadruplex Potassium Probes for Anchoring at the Langmuir Monolayer and Cell Membrane

**DOI:** 10.3390/s18072201

**Published:** 2018-07-09

**Authors:** Angelika Świtalska, Anna Dembska, Agnieszka Fedoruk-Wyszomirska, Bernard Juskowiak

**Affiliations:** 1 Faculty of Chemistry, Adam Mickiewicz University, Umultowska 89b, 61-614 Poznan, Poland; aniojka@amu.edu.pl (A.D.); juskowia@amu.edu.pl (B.J.); 2 Institute of Bioorganic Chemistry, Polish Academy of Sciences, Noskowskiego 12/14, 60-704 Poznan, Poland; agaw@ibch.poznan.pl

**Keywords:** cholesterol anchor, G-quadruplex, FRET, pyrene, Langmuir monolayer, living cell membrane, potassium sensing

## Abstract

The purpose of the present work was to design, synthesize and spectrally characterize cholesterol-anchored fluorescent oligonucleotide probes (Ch(F-TBA-T), Ch(py-TBA-py)), based on G-quadruplexes, which were able to incorporate into a lipid structure (Langmuir monolayer, living cell membrane). The probes, based on the thrombin-binding aptamer (TBA) sequence, were labeled with fluorescent dyes which enabled simultaneous monitoring of the formation of G-quadruplex structures and visualization of probe incorporation into the cellular membrane. The combinations of fluorophores used included fluorescence resonance energy transfer (FRET) and excimer emission approaches. The structural changes of the probes upon binding with K^+^ or Na^+^ ions were monitored with fluorescence techniques. These systems showed a very high binding preference for K^+^ over Na^+^ ions. The use of confocal fluorescence microscopy indicated successful anchoring of the cholesterol-bearing fluorescent probes to the living cell membrane. These structurally simple cholesterol-based fluorescent probes have good potential for opening up new and exciting opportunities in the field of biosensors; e.g., in vivo detection of K^+^ ions.

## 1. Introduction

The structure and function of biological membranes are largely controlled by their lipid composition, and particularly by cholesterol, which represents up to 40% of the lipids in plasma membranes [1,2]. Since the pioneering works of Letsinger [3,4] and Stein [5], a large number of oligonucleotides have been modified with cholesterol to enhance their biological activity [6].

On the other hand, functionally designed lipid-anchored DNA oligonucleotides have significantly promoted many important developments in fields such as nanobiotechnology [7,8,9], cell biology [9,10,11], and the development of therapeutic strategies in medicine [12,13]. To precisely control the cellular behaviors in these applications, an improved understanding of the physicochemical properties of cell membranes as well as their interactions with DNA is required. Amphipathic oligonucleotides have been heavily exploited to mimic membrane association processes in natural systems; however, the interactions between biomembranes and G-quadruplexes are very poorly understood. There is much debate about the existence of G-quadruplexes in cells, although G-quadruplexes have recently been detected in human cells using a structure-specific antibody [14].

G-quadruplexes are unique higher-order structures formed by G-rich nucleic acid sequences, based on stacked arrays of G-quartets, and connected by Hoogsteen type cyclic hydrogen bonding. Formation of G-quadruplexes requires runs of guanine on one, two or four strands, resulting in linear (four strand), or folded (one or two strand) structures [15]. It is also known that a G-quadruplex has a channel at its center with a diameter that correlates well with the ionic radius of K^+^ (1.3 Å) and Na^+^ (1.16 Å); hence, physiological buffer conditions favor their formation [16]. In particular, a K^+^ ion can be located in the cavity between two adjacent G-tetrads of a G-quadruplex due to its appropriate size, coordinated by eight carbonyl oxygen atoms from the G-tetrads. The coordination contributes to K^+^ having the highest efficiency among all alkali cations for stabilizing G-quadruplex, which confers the selectivity of some G-quadruplex DNAs for K^+^ [17]. Sensitive and selective detection of K^+^ is essential to biomedical diagnosis. However, it is a challenge to selectively determine the extracellular K^+^ concentration, owing to the presence of the large excess of sodium ions (Na^+^) in physiological conditions. In serum, the normal concentration of K^+^ is 3.5–5.3 mM, whereas that of Na^+^ is 135–148 mM [18]. Recently, there have been more and more studies using G-quadruplex DNAs as sensing elements for K^+^ detection. The oligonucleotide-based potassium probes have already been developed in our group, and by others, mainly as sensors for potassium in bulk solution [19,20,21,22,23]. In our group [24], oligonucleotides modified with triethyleneglycol-cholesteryl (TEG-cholesteryl) have been developed.

In this paper, we made use of cholesterol-anchored fluorescent probes (Ch(F-TBA-T), Ch(py-TBA-py)) based on G-quadruplexes for spontaneous anchoring into the hydrophobic interior of lipid structures (Langmuir monolayer, living cell membrane). Fluorescent oligonucleotide probes have a cholesterol moiety (Ch) attached to the 5′ end of a thrombin-binding aptamer (TBA) sequence via a triethylene glycol (TEG) spacer. Cholesterol-based anchoring molecules are also found in eukaryotic membranes, and these molecules can be incorporated into lipid membranes without disrupting the bilayer structure [25]. The Ch(F-TBA-T) probe was labeled with FAM (carboxyfluorescein) and TAMRA (carboxytetramethylrhodamine) dyes, and the Ch(py-TBA-py) probe with pyrene groups at both termini. As a reference, we carried out experiments with analogous probes without the cholesterol group (F-TBA-T, py-TBA-py). 

We present here the spectral properties and G-quadruplex folding ability of these fluorescent oligonucleotide probes at the Langmuir monolayer interface, which mimics a biomembrane surface. Since biological membranes are difficult to study in vivo, much of our understanding of their complex behavior comes from model surfactant bilayer systems such as the Langmuir monolayer. To mimic cellular membranes we used a cationic DODAB (dimethyldioctadecylammonium bromide) amphiphile. Fluorescence spectra of probes at the air/aqueous subphase interface were recorded using a Langmuir trough and spectrofluorimeter equipped with a fiber optic accessory. We utilized UV, fluorescence and circular dichroism (CD) spectroscopy techniques to monitor the folding and spectral properties of G-quadruplexes formed by the designed probes in bulk solutions. Thermal denaturation experiments were also carried out to evaluate the stability of the G-quadruplexes. Finally, a cell-based study was performed to assess the performance of potassium ion sensing probes in live HeLa cells. We loaded two probes into HeLa cells to evaluate their ability to fold into G-quadruplex structures after anchoring in membrane of living cells.

## 2. Experimental Section

### 2.1. Materials

**Scheme 1:**
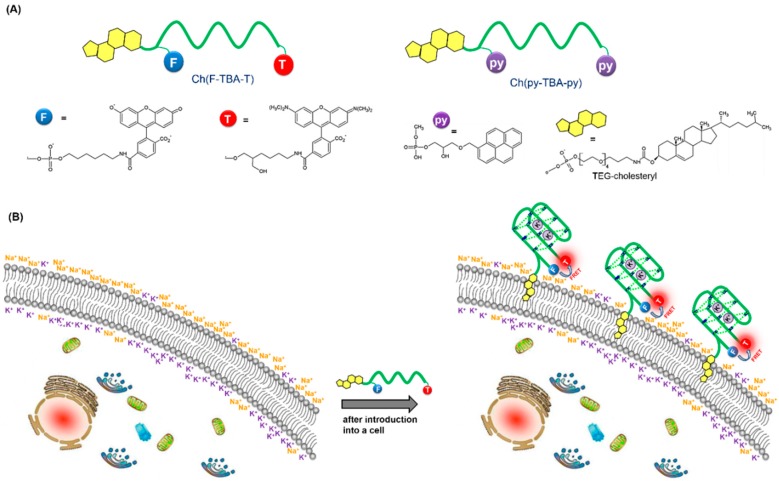
Structure of the cholesterol−anchored fluorescent probes (**A**), working principle of cell-anchored cholesterol−fluorescent probes (**B**).

The oligodeoxyribonucleotide-based probes, (F-TBA-T, py-TBA-py, Ch(F-TBA-T) and Ch(py-TBA-py)) were synthesized by Eurogentec (Liège, Belgium), and purified by reversed phase HPLC. Their identities were confirmed by MALDI-TOF MS. Potassium and sodium binding preferences were carefully tuned by selection of a quadruplex-forming oligonucleotide. To avoid problems with tetraplex polymorphism, we chose the 15-mer oligonucleotide with the sequence of d(GGTTGGTGTGGTTGG), known as a thrombin-binding aptamer (TBA) [26]. In order to form a suitably flexible structure with an optimum ability to incorporate into the hydrophobic part of the lipid membrane, and to form G-quadruplex, the oligonucleotide has the cholesterol-anchoring moiety, which is linked to the deoxyribose via a triethyleneglycol (TEG) spacer (Scheme 1A). The py-TBA-py probe is labeled with pyrene groups at the 5′ and 3′ ends. In turn, the Ch(py-TBA-py) probe contains an additional T base at the 5′ end, and a cholesterol moiety attached to the 5′ end of the oligonucleotide. In the case of probe F-TBA-T, the basic TBA sequence was extended by 4 bases (ATTT) on the 3′ end, which improved performance of the F-TBA-T probe as result of protection from contact quenching caused by the dye–dye interactions [20]. In the Ch(F-TBA-T) probe, the TBA sequence was extended by 4 bases at both 5′ and 3′ ends, TTTA and ATTT, respectively. This probe comprises a cholesterol moiety attached to the 5′ end of the oligonucleotide, TAMRA is attached to the 3′ end, and FAM is placed internally via dT modification. The primary sequences and names of the fluorescent oligonucleotides are given in Table 1. Concentrations of probes were determined using molar absorptivities at 260 nm, since at this wavelength absorbance is not affected by folding into quadruplex [27]. The contribution of label absorbance at 260 nm was assumed to be ca. 20% of the absorbance of long-wavelength bands for the labels [28]. The molar absorptivities for DNA parts were calculated by the nearest-neighbor method, from published values of E_260_ for monomer and dimer DNA [29].

All other reagents were purchased from Sigma Aldrich (St. Louis, MO, USA) and were used as received. Potassium chloride (KCl), sodium chloride (NaCl) and dioctadecyldimethylammonium bromide (DODAB) were of extra pure quality. The buffer used in this work was 10 mM Tris adjusted to ca. pH 7.5 with acetic acid and contained 1 mM EDTA (TAE buffer). High-purity water (Polwater, Kraków, Poland) was used throughout. In studies concerning the Langmuir monolayer, Tris buffer, without EDTA addition, adjusted to required pH by acetic acid was used (TA buffer).

### 2.2. Methods

#### 2.2.1. UV-Vis Absorption Studies

UV-Vis spectra and thermal denaturation profiles were obtained with a Cary 100 UV-Vis spectrophotometer with temperature controlled Peltier accessory (Agilent Technologies, Mulgrave, Australia). Absorption spectra of the oligonucleotide probes were recorded in the spectral range of 200–800 nm at 25 °C. Thermal denaturation profiles were measured at a heating rate of 1 C/min, with absorbance monitoring at 260 nm and 295 nm as a function of temperature. Determination of melting temperatures was carried out using a method similar to that previously published [27,30].

#### 2.2.2. Fluorescence Spectra

All measurements were performed on a Cary Eclipse spectrofluorimeter (Agilent Technologies, Mulgrave, Australia) with 5 nm excitation and emission slits, and were carried out using 0.4 × 1 cm quartz cuvettes, containing 1 mL of sample solution. Fluorescence emission spectra for pyrene-labeled probes were recorded in the 350–650 nm range, with the excitation wavelength of 340 nm. In turn, fluorescence emission spectra for FRET-labeled probes were recorded in the 500–800 nm spectral range, with the excitation wavelength of 490 nm for FAM and 560 nm for direct excitation of TAMRA. A typical investigation of metal cation effect in the concentration range of 0–0.15 M consisted of successive additions of small portions (0.1–50 μL) of a concentrated solution of 3 M KCl or 3 M NaCl salt, followed by stirring and thermal equilibration. Studies of the metal cation effect were carried out at 25.0 °C and 36.6 °C. 

FRET efficiency that depended on G-quadruplex formation was evaluated using the ratio of the fluorescence intensity of the TAMRA acceptor excited by 490 nm to that of the FAM donor: (ratio) = F_583_/F_520_. The monomer/excimer (m/e) ratio (F_490_/F_380_), calculated by comparing the fluorescence intensity of the first monomer peak (typically ~380 nm) with that of the excimer band (generally ~490 nm), is a relative indicator of the extent of excimer formation, and thus G-quadruplex folding.

#### 2.2.3. CD Spectroscopy

Circular dichroism (CD) measurements were carried out on a Jasco J-820 Spectropolarimeter (Jasco, Tokyo, Japan) equipped with a PTC-423L temperature controller. Spectra were recorded at room temperature approximately 30 min after sample preparation in 1 cm path length quartz cells using 1 µM solution of a probe in 10 mM TAE buffer (pH 7.5) and potassium or sodium salt. A typical investigation of metal cation effect in the concentration range of 0–0.15 M consisted of successive additions of small portions (0.1–50 μL) of a concentrated solution of 3 M KCl or 3 M NaCl salt, followed by stirring and thermal equilibration. The effect of temperature was evaluated by heating the samples from 10 °C to 90 °C (1 °C/min) and recording the CD spectra at specific intervals of 5 °C degrees after thermal equilibrium was attained. The CD spectra were obtained by taking the average of three scans in the range of 340–220 nm, with a scan rate of 200 nm/min. The scan of the corresponding buffer solution was subtracted from the average scan for each sample. 

#### 2.2.4. Measurements of π-A Isotherms and Fluorescence Spectra at the Monolayer Interface

A computer-controlled film balance system (Langmuir trough small, KSV NIMA, Espoo, Finland) equipped with a platinum Wilhelmy plate pressure sensor was used to obtain the surface pressure–area (*π*-A) isotherms for the DODAB (dimethyldioctadecylammonium bromide) monolayer at the air–water interface, and to maintain required surface pressure of the monolayer upon recording fluorescence spectra of the probes adsorbed at the monolayer interface. The temperature of the subphase was kept at 25.0 °C ± 0.2 °C by means of a water circulation bath. The monolayer was obtained by spreading the solution of DODAB in chloroform (1 mM) onto a subphase containing buffer and other reagents (fluorescent probes and/or salts) using a microsyringe. Compression experiments started after a 15-min incubation to evaporate the organic solvent. A symmetric compression with a constant barrier speed of 5 cm^2^/min was used. The trough size and volume were 195 × 50 mm and 50 mL respectively.

Fluorescence measurements of monolayer-adsorbed probes were carried out using a system consisting of a Langmuir trough and Cary Eclipse spectrofluorimeter equipped with a fiber optic accessory (Agilent Technologies, Mulgrave, Australia) shown in Scheme S1 in the electronic supplementary information (ESI). The DODAB monolayer was formed on the subphase containing the probe at a strand concentration of 8.3 nM (the charge ratio of the DODAB:probe was 1:1). 

Fluorescence emission spectra for pyrene-labeled probes (py-TBA-py, Ch(py-TBA-py)) were recorded in the 350–650 nm range, with the excitation wavelength of 330 nm. In turn, fluorescence emission spectra for FRET-labeled probes (F-TBA-T, Ch(F-TBA-T)) were recorded in the 500–800 nm spectral range, with the excitation wavelength of 470 nm for FAM and 550 nm for TAMRA. The background spectra were first recorded prior to DODAB spreading, and then after monolayer formation during different experimental conditions. The effect of surface pressure on fluorescence spectra was tested after stepwise compression of the monolayer from π = 0 to 20 mN/m. A 10-min delay followed each compression step to allow relaxation of the monolayer. The fluorescence spectra exhibited reproducible characteristics within at least 60 min in the compressed state.

#### 2.2.5. Introduction of the Probe into Membrane of Living Cell for Fluorescence Imaging Experiment

HeLa were seeded at a density of 1.2 × 10^5^ cells per well in 4-chamber glass-bottom cell culture dishes (Grenier Bio-One, Kremsmünster, Austria) and cultured in RPMI 1640 antibiotic-free medium (Sigma, Kawasaki, Japan) supplemented with 10% (*v*/*v*) fetal bovine serum (FBS) (Gibco), and 1% RPMI 1640 vitamin solution (Sigma, Kawasaki, Japan) at 37 °C under a 5% CO_2_ atmosphere. After one day, the cells reached the appropriate density (80–90% confluence) and were placed in fresh RPMI 1640 medium without supplements. Then the Ch(F-TBA-T) probe was added to the culture medium to obtain the final concentration,50 nM or 200 nM. The visualizations were done after 24 h of treatment. The three other sample sets with Ch(F-TBA-T) (50 nM) were prepared 3.5 h, 2.0 h and 30 min. before visualization. The positive control was HeLa cells treated with the F-TBA-T probe (50 nM) added to the culture medium 24 h before visualization. The negative control was untreated cells. Before fluorescence confocal microscopy analysis, cells were washed twice with phosphate buffered saline (PBS) and placed in FluoroBright Live Cell Fluorescence Imaging Medium. In experiments using Hoechst 33342 (Sigma, Kawasaki, Japan), the dye was applied to medium in a final concentration of 5 µg/mL and images were taken after 15 min.

#### 2.2.6. Fluorescence Imaging Experiments

Imaging was carried out with a confocal laser fluorescence inverted microscope using a Spectral Confocal Microscopy TCS SP5 II Leica system equipped with a white light laser (470–670 nm) and a 405 laser, and an environmental cell culture chamber that provided controlled conditions of temperature, CO_2_ saturation and humidity. Sequentially scanned live-cell images were collected using a Plan Apo 63 × 1.4 NA oil-immersion objective. Leica LAS AF SP software was used for control of image processing and fluorescence analysis. The fluorescence imaging was taken using excitation/emission (ex/em) wavelengths as follows:

(a) FAM (fluorescein) channel, ex/em range = 480/510–540 nm.
(b) TAMRA channel, ex/em range = 560/595–630 nm.
(c) FRET channel, ex/em range = 480/595–630 nm.
(d) Hoechst channel, ex/em range = 405/480–500 nm.

## 3. Results and Discussion

### 3.1. Design and Working Principle of Cholesterol-Anchored Fluorescent Oligonucleotide Probes 

The model cholesterol-anchored fluorescent oligonucleotide probes for potassium sensing at the cell membrane interface are illustrated in Scheme 1. The amphiphilic lipid−DNA probe consists of a hydrophobic cholesterol moiety, hydrophilic DNA strand, and fluorophores attached to DNA (Scheme 1A). The Ch(F-TBA-T) probe was modified with FAM and TAMRA, whereas the Ch(py-TBA-py) probe was modified with pyrene groups, at the 5′- and 3′-termini of the oligonucleotide, respectively. Due to the phospholipid bilayer structure of the cell membrane, lipid−DNA probes can be directly anchored on the membrane through the hydrophobic interaction between the cholesterol moiety and the phospholipid. Thus, anchoring DNA makes use of the naturally occurring membrane constituent, consequently eliminating the risk of side effects induced by chemically reactive lipid headgroups incorporated in membrane constituents [25].

Intramolecular folding of a flexible single-stranded DNA molecule into a compact G-quadruplex leads to closer proximity of its 5′ and 3′ ends. Thus, labeling both ends of the DNA strand with suitable fluorophores enables monitoring of the quadruplex-formation process as result of the fluorescent signal generation. In our research, we use two types of fluorescent approaches: (i) based on the fluorescence resonance energy transfer (FRET); and (ii), switching of monomer–excimer emission of pyrene fluorophores. FRET between two molecules is an important physical phenomenon, where transfer of energy occurs from an excited donor fluorophore to a suitable acceptor fluorophore. The basic requirements for the FRET to occur are: (i) sufficient overlap between the absorption band of acceptor and the fluorescence band of donor; and (ii), both the donor and the acceptor molecule must be in close proximity, in the order of 1–10 nm. The interference of solvent or other macromolecules has little effect on FRET efficiency [31,32]. The advantages of the pyrene probes are the high Stokes shift between monomer and excimer emission, and their relatively long lifetime (30–60 ns) compared to the lifetime of the autofluorescence background (about 8 ns) [32,33,34].

In the first strategy, an oligonucleotide-bearing thrombin binding aptamer sequence was end-labeled with fluorescent groups (FAM and TAMRA) to give FRET probe—Ch(F-TBA-T). The Ch(F-TBA-T) probe additionally has a cholesterol anchor at the 5′ end. FAM and TAMRA dyes were chosen as donor and acceptor, respectively. The probe exhibited the ability to fold into the quadruplex structure and to bind metal cations (Na^+^ and K^+^). As shown in Scheme 1B, before anchoring into membrane (in an unbound state), Ch(F-TBA-T) possesses spatially separated termini and exhibits an unperturbed fluorescence spectrum dominated by FAM (donor) emission. Upon incorporation into the membrane, and in the presence of K^+^ (Na^+^) ion, the quadruplex structure is developed that enables fluorophores to be arranged in close proximity causing alteration of their fluorescence spectra (quenching of the FAM band and enhancement of the TAMRA emission). 

The second type of probe (Ch(py-TBA-py)) also possesses cholesterol moiety and can be anchored into the cell membrane but uses the pyrene excimer emission for the transduction of the cation-binding event. After G-quadruplex structure formation, in which two pyrenes are located in close proximity, an excimer emission can appear. This excimer exhibits a broad red-shifted emission band near 480 nm, in contrast with the structured emission of the pyrene monomer at 380 and 400 nm. 

Two reference probes were also designed, which lacked the cholesterol anchor but preserved other structural components (TBA recognition oligonucleotide and pyrene or FRET labels).

### 3.2. Spectral Properties of the Fluorescent Probes in Solution

The spectral properties of fluorescent oligonucleotides were first investigated in buffer solution in order to verify their abilities to generate fluorescence signal upon G-quadruplex formation. All probes were tested in sample solutions containing metal cations at concentrations corresponding to the intra- and extra- cellular conditions. 

The FRET-probes (F-TBA-T, Ch(F-TBA-T)) exhibited absorption bands at 260 nm, 494 nm, and 560 nm (Figure S1A,B ESI), and the pyrene-labeled probes (py-TBA-py, Ch(py-TBA-py)) exhibited absorption bands at 260 nm, 326 nm, and 340 nm (Figure S1C,D, ESI), as expected for dual-labeled oligonucleotides with FAM-TAMRA and py-py labels, respectively. The long-wavelength absorption bands are characteristic of FAM and TAMRA (494 nm and 560 nm, respectively) and pyrene groups (325 nm, 340 nm), but the band at 260 nm should be ascribed mainly to the absorption of nucleobases. It should be noted that very similar spectra were obtained for all probes studied in the absence as well as presence of K^+^ and Na^+^ ions, irrespective of whether they contained cholesterol moiety or not. This is not surprising since cholesterol has a low molar absorption band at 260 nm (ε ~ 100 [35]). 

Thermal stability and kinetics of G-quadruplex folding for cholesterol-containing fluorescent probes were evaluated in the presence of K^+^ (100 mM) or Na^+^ (100 mM) ions using the denaturation profiles recorded with absorbance at 295 nm. The obtained melting profiles are shown in Figure S2, ESI. Temperatures at half transition for the all probes in the presence of K^+^ and Na^+^ ions can be found in Table 2. The literature reports melting temperatures of TBA in the presence of Na^+^ and K^+^ of 24.0 °C and 53.0 °C, respectively [36,37]. The attached pyrene moieties to the 5′ and 3′ ends of the py-TBA-py probe did not significantly affect the melting temperature (55.0 °C). However, the attachment of FAM/TAMRA dyes, and the increase in the length of the oligonucleotide chain (ATTT spacer), significantly affected the Tm in the case of the F-TBA-T/K^+^ complex. The melting temperature for F-TBA-/K^+^ dropped to 38.0 °C. Interestingly, melting temperatures for the F-TBA-T/Na^+^ and py-TBA-py/Na^+^ systems were almost unaltered compared with that for TBA.

A possible explanation of lower Tm for the F-TBA-T/K^+^ system may involve the more hydrophilic character of this G-quadruplex compared with the native TBA. Both the dye groups and the four-nucleotide spacer introduce additional charges to the probe molecule. Mergny et al. [38] studied the effect of the nucleobase spacer on the stability of tetraplex structures of G-rich oligonucleotides. They showed that an increase in the number of nucleotides in the spacer led to the decrease in thermal stability of the tetraplex structure, which concurs with our observations. It should be noted that the presence of cholesterol moiety, which increases the hydrophobic character of probes, significantly stabilized the G-quadruplexes (Table 2).

**Figure 1:**
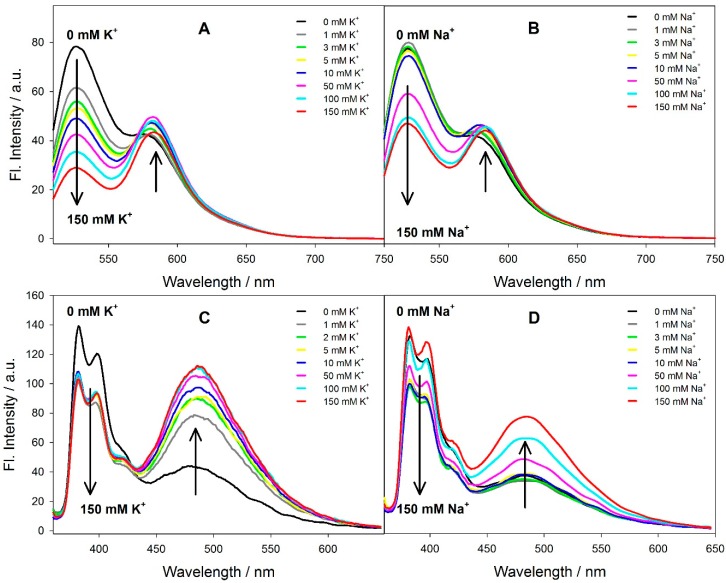
Potassium and sodium ions effect on the fluorescence spectra of 0.2 µM Ch(F-TBA-T) ((**A**,**B**), respectively) with *λ*_ex_ = 490 nm, and Ch(py-TBA-py) ((**C**,**D**), respectively) with *λ*_ex_ = 340 nm. Conditions: 10 mM TEA buffer (pH 7.5), KCl and NaCl concentration: 1, 3, 5, 10, 50, 100, 150 mM.

The fluorescence spectra of the Ch(F-TBA-T) probe, and its analogue without cholesterol (F-TBA-T), in the presence of K^+^ ion (0–150 mM) are shown in Figure 1A and Figure S3A, ESI, respectively. Spectra related to Ch(F-TBA-T)/Na^+^ and F-TBA-T/Na^+^ are shown in Figure 1B and Figure S3B, ESI, respectively. The fluorescence band observed at λ_em_ = 520 nm is characteristic of the FAM emission and the band at λ_em_ = 583 nm is typical for the TAMRA fluorophore. In the unfolded form of both probes (no metal cations), little FRET is expected since the average distance between the donor and the acceptor (ca. 80 Å) exceeds the critical radius (ca. 50 Å). Folding of the probe into G-quadruplex with increasing concentration of cations brings these two fluorophores close enough for energy transfer to be efficient. For both probes an increase in K^+^ ion concentration (0→ 150 mM) caused considerable quenching of the FAM emission band at λ_em_ = 520 nm whereas the intensity of the TAMRA band λ_em_ = 583 nm slightly increased (Ch(F-TBA-T), Figure 1A) or decreased (F-TBA-T, Figure S3A, ESI). It should be noted that the FAM and TAMRA fluorescence bands for the F-TBA-T probe (Figure S3A,B, black line, ESI) possess comparable intensity in the absence of metal ions. The relative high intensity of TAMRA emission in the extended form (no K^+^ ion) of the F-TBA-T probe suggested that this probe might have a shrunken conformation (less than 60 Å rather than its extended one), although the direct excitation of TAMRA acceptor could not be neglected. In turn, the extension of the Ch(F-TBA-T) probe (four bases) changes the donor-acceptor distance, which affects the FRET efficiency. In the absence of K^+^, fluorescence intensity of the FAM emission band increases, while the relative fluorescence intensity of TAMRA band decreases (Figure 1A). Contrary to potassium, sodium ions exert a much smaller effect on the fluorescence spectra of the probes. Folding of the G-quartet oligonucleotides in the presence of sodium ions was visible only at 50 mM for the Na^+^ ion but at 1 mM in the case of K^+^ ion. It should be pointed out that upon direct excitation at 560 nm, all probes exhibited a substantial decrease in TAMRA fluorescence with an increase in cation concentration (Figure S4, ESI). Some of the spectral properties for the F-TBA-T and Ch(F-TBA-T) probes have been described elsewhere [39].

**Figure 2:**
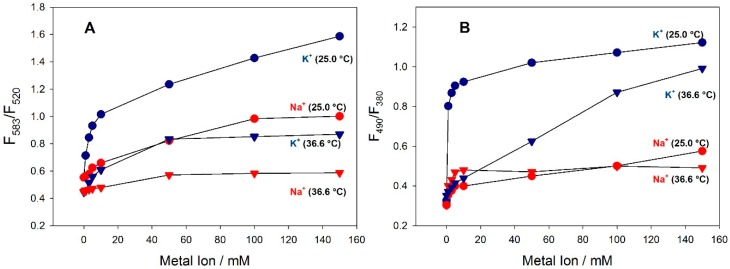
Fluorescence intensity ratio of Ch(F-TBA-T) (F_583_/F_520_) (**A**) and Ch(py-TBA-py) (F_490_/F_380_) (**B**) systems plotted against K^+^ or Na^+^ concentration at 25 °C and 36.6 °C.

Figure 2A shows variation of the FRET efficiency expressed as a fluorescence intensity ratio (F_583_/F_520_) for Ch(F-TBA-T) plotted against the concentration of K^+^ (blue symbols) and Na^+^ (red symbols) at 25.0 °C (circles) and 36.6 °C (triangles). The fluorescence intensity ratio (F_583_/F_520_) of F-TBA-T (probe without cholesterol moiety) is shown in Figure S5A, ESI. For both temperatures, an increase in the FRET signal was observed in the range of 1–10 mM for K^+^ and in the range of 10–150 mM for Na^+^ ions, which is consistent with the TBA binding affinities of these cations. The values of the fluorescence ratio (F_583_/F_520_) for F-TBA-T are larger than those for Ch(F-TBA-T) (compare Figure 2A and Figure S5A, ESI). This is reasonable when considering that two dyes in the F-TBA-T probe should be closer, since the length of a 19-meric TBA is 80 Å. However, titration with sodium and potassium cations at 36.6 °C decreased the FRET signal by ca. 50% in both cases. Moreover, Ch(F-TBA-T) also exhibited good binding selectivity for the K^+^ over the Na^+^ ion, both at 25.0 °C and at 36.6 °C (Figure 2A). The F-TBA-T probe exhibited similar spectral changes (Figure S5A, ESI). A plateau region was observed for most plots at higher cation concentrations. 

The difficulty in the monitoring K^+^ level in extracellular conditions comes from the coexistence of an excess of sodium ions (Na^+^), thus small variations in K^+^ concentration (around 5 mM K^+^) should be monitored in the presence of 140 mM Na^+^ concentration. Because of the high content of Na^+^, the efficient formation of potassium-quadruplex complex requires significant binding preferences for K^+^ over Na^+^. Figure S6, ESI shows the fluorescence spectra of Ch(F-TBA-T) in the presence of 150 mM Na^+^ titrated with K^+^ ions in the 1–20 mM concentration range at 25.0 °C (Figure S6A, ESI) and 36.6 °C (Figure S6B, ESI). Figure S7, ESI shows the potassium calibration graphs plotted with a ratio of F_583_/F_520_) under the same conditions. The probe showed a dynamic range for K^+^ detection of 2–10 mM in the presence of 150 mM Na^+^, which is a clinically important concentration region of K^+^ under an extracellular conditions.

Development of a second type of fluorescent probe exploited the pyrene excimer emission for the transduction of the cation-binding event. The fluorescence spectra of Ch(py-TBA-py) and py-TBA-py (λ_ex_ = 380 nm) in the presence of K^+^ (0–150 mM) are shown in Figure 1C and Figure S3C, ESI respectively; and those in the presence of Na^+^ ion (0–150 mM) are shown in Figure 1D and Figure S3D, ESI, respectively. A typical excimer emission characteristic of pyrene with a broadband at ca. 490 nm is observed for both probes in the presence of K^+^ ions, and only for the Ch(py-TBA-py) probe in the presence of Na^+^. Contrary to potassium, sodium ions exerted much smaller effect on the fluorescence spectrum of the py-TBA-py probe. Folding of the py-TBA-py in the presence of Na^+^ ions only led to quenching of pyrene monomer emission, without the concomitant increase in excimer emission at longer wavelengths. It should be noted that the fluorescence spectra of both Ch(py-TBA-py) and py-TBA-py probes in the absence of metal ions exhibit monomer bands with defined peaks at 380 nm and 400 nm, and a weak shoulder at 490 nm. The weak band at 490 nm proves that the pyrene moieties interact with each other when probes are in a random coil conformation [33]. The potassium ion gave a positive response at just 1 mM, and the sodium ion gave detectable excimer emission for concentrations of Na^+^ ions exceeding 50 mM, which is consistent with the results for FRET-labeled probes.

The monomer/excimer (m/e) ratio, calculated by comparing the fluorescence intensity (or quantum yield) of the first monomer peak (typically ~380 nm) with the excimer band (generally ~490 nm), is a relative indication of the extent of excimer formation, and therefore a measure of spatial proximity between two pyrene moieties. Figure 2B shows the variation of the excimer formation efficiency (F_490_/F_380_) for Ch(py-TBA-py) plotted against the concentration of K^+^ (blue symbols) and Na^+^ (red symbols) at 25.0 °C (circles) and 36.6 °C (triangles). Appropriate plots for py-TBA-py (probe without cholesterol moiety) are shown in Figure S5B, ESI. Comparison of the profiles of all plots for py-TBA-py and Ch(py-TBA-py) indicates that: (i) potassium is more effective and produces excimer signal at lower concentration than sodium ion; (ii) the excimer formation efficiency finally attained is higher for K^+^ than for Na^+^; and (iii), the F_490_/F_380_ ratio is slightly smaller at 36.6 °C than at 25.0 °C. One should remember, however, that additional factors may affect fluorescence signal; for example, quenching/dequenching processes of FAM and TAMRA fluorophores, pyrene/DNA interactions and presence of a cholesterol group may operate upon the folding of oligonucleotide, and such phenomena also affect the FRET and excimer signals.

**Figure 3:**
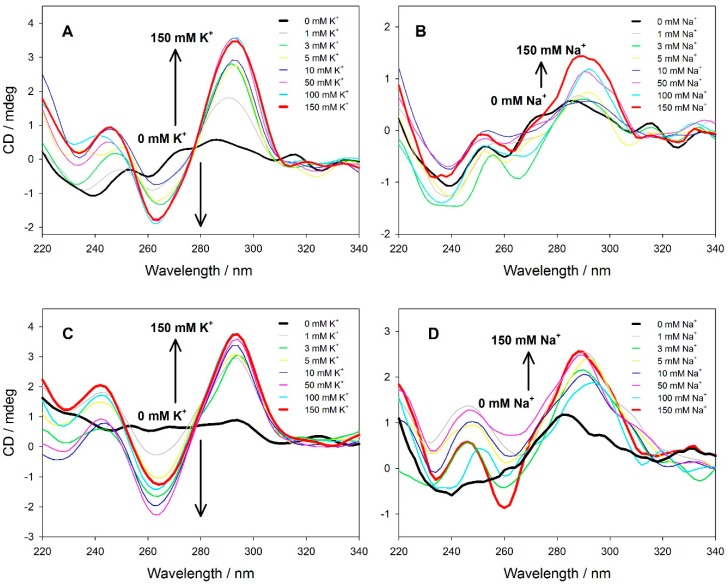
Effect of potassium and sodium ions on the CD spectra of 1 µM Ch(F-TBA-T) (**A**,**B**) and Ch(py-TBA-py) (**C**,**D**) probes. Conditions: 10 mM TEA buffer (pH 7.5), KCl and NaCl concentration: 1, 3, 5, 10, 50, 100, 150 mM.

More detailed studies on G-quadruplex formation with cholesterol-bearing fluorescent probes were carried out using CD spectroscopy. A thrombin-binding aptamer (TBA) used in this study (Table 1) is known to form only a chair-type anti-parallel intramolecular G-quadruplex structure in the presence of metal cations [40], and such quadruplex formation is readily identified through the positive bands around 295 nm and 240 nm, and the negative band around 270 nm in the CD spectrum. Among the various monovalent cations, potassium ions stabilize the TBA quadruplex the most effectively; consequently, the CD spectrum of the TBA–potassium complex exhibits greater band intensities compared with those of other TBA–cation complexes, for example, Na^+^ [41]. The representative CD spectra for Ch(F-TBA-T) and Ch(py-TBA-py) titrated with KCl and NaCl (0–150 mM) are shown in Figure 3A–D, respectively. CD spectra for the reference probes without cholesterol moiety (F-TBA-T, py-TBA-py) in the same environmental conditions are shown in Figure S8, ESI. Weak CD peaks are observed for both probes in the absence of KCl, whereas the addition of salt (1–150 mM) causes an appearance of the characteristic G-quadruplex signature—a negative band at 265 and positive bands at 240 and 295 nm (Figure 3A,C), similar to that observed for the F-TBA-T/K^+^ and py-TBA-py/K^+^ complexes without cholesterol moiety (Figure S8A,C, ESI). The intensity of the CD bands rises as the salt concentration increases up to 10 mM KCl. Further addition of salt causes only minor effects on the CD spectra of both probes, indicating that 10 mM concentration of KCl is sufficient for nearly complete probe formation of quadruplex structures.. CD spectra recorded for all probes (Figure 3B,D and Figure S8B,D, ESI) showed less pronounced spectral changes upon the addition of Na^+^ ions (0–150 mM). A structural change from a random coil to a chair-type quadruplex structure (the negative band near 265 nm and the positive band at 290 nm) was detectable for concentrations of Na^+^ ions exceeding 100 mM. The presence of Na^+^ cations causes small changes in the CD spectra because TBA forms a weak complex with Na^+^ ions as a consequence of the difference in binding mode between potassium and sodium ions, in accordance with data in the literature [41].

This result suggested that all probes underwent a conformational change in the presence of K^+^ and Na^+^ to an intramolecular tetraplex from the random structure. However, the CD intensity of the K^+^ induced G-quadruplex is much higher than that induced by the sodium ion. The CD patterns for cholesterol-anchored fluorescent probes are very similar to those observed for probes without the cholesteryl group. All these data suggest that attachment of the FAM/TAMRA labels, pyrene groups and cholesterol anchor have negligible effect on the folding properties of TBA oligonucleotide.

### 3.3. Fluorescent Probes at the Water/Monolayer Interface

To demonstrate the feasibility of using cholesterol-containing fluorescent oligonucleotide probes for potassium sensing in living cells, we have carried out preliminary experiments with a Langmuir monolayer of DODAB. The cholesterol-linked probes were soluble in all physiologically relevant solutions and could interact with the Langmuir monolayer via hydrophobic interaction between the cholesterol moiety and the membrane, or via electrostatic interaction between the positively charged DODAB head groups and the negatively charged phosphate groups of DNA. 

Oligonucleotides possess negative charges and, being linear, polyelectrolytes can undergo adsorption on the monolayer of cationic surfactants at the air/water interface due to electrostatic attraction forces [42,43,44,45,46,47]. Our earlier surface pressure—area study showed that interactions of the unlabeled G4 DNA oligonucleotide did not occur with the zwitterionic DPPC (dipalmitoylphosphatidylcholine) monolayer but the π -A isotherm of the cationic Langmuir film (DODAB) shifted to lower molecular areas in the presence of G4 DNA, indicating electrostatic interactions between these two components. However, probes equipped with the cholesterol anchor are expected to incorporate into the monolayer due to the hydrophobic interactions. Nevertheless, the extent of probe-monolayer interactions, which is reflected by the magnitude of surface pressure (area) change for the compressed monolayer, is governed by charge density of both the monolayer head group region and the counter anion layer. This anionic layer is modified by the presence of DNA molecules. The number of DNA strands interacting with the monolayer is dependent on the concentration and the nature of other counter anions in a subphase (buffer, added salts). The subphase containing 10 mM TA buffer allowed observation of the oligonucleotide effect on the DODAB monolayer compression at equimolar concentration of DNA and DODAB charges.

**Figure 4:**
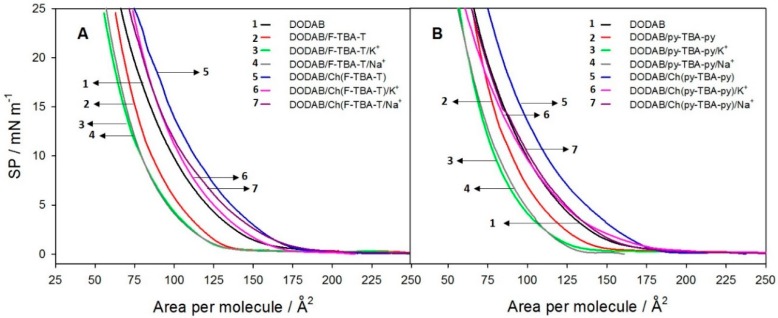
The π-A isotherms recorded for DODAB monolayer on a subphase containing 8.3 nM F-TBA-T and Ch(F-TBA-T) probes. (**A**), 8.3 nM py-TBA-py and Ch(py-TBA-py) probes. (**B**) in the absence and presence of potassium or sodium ions. Subphase contained 10 mM TA buffer, pH = 7.5.

Figure 4 shows π-A isotherms of the DODAB monolayer recorded on the subphase containing 8.3 nM F-TBA-T or Ch(F-TBA-T) probes (Figure 4A) and py-TBA-py or Ch(py-TBA-py) probes (Figure 4B) in the absence and in the presence of potassium or sodium ions. The isotherms recorded on the F-TBA-T- or py-TBA-py–containing subphases (line 2, Figure 4A,B) are shifted into the lower surface area per molecule, when compared with the reference isotherms recorded on the subphase containing only 10 mM TA buffer (line 1, Figure 4A,B) that indicates adsorption of DNA strands on the subphase/monolayer interface. Addition of metal cations (K^+^ and Na^+^) that are known to promote G-quadruplex formation further affected π -A isotherms, in agreement with results reported previously for similar systems [46,47]. The addition of metal cations (potassium, line 3 and sodium, line 4; Figure 4A,B) further affected π -A isotherms by inducing G-quadruplex folding that resulted in a more compact monolayer formation, also in agreement with results reported previously for similar systems [46,47]. In contrast, isotherms recorded for DODAB monolayers in the presence of cholesterol-type probes (Ch(F-TBA-T) and Ch(py-TBA-py)) in the subphase (line 5, Figure 4A,B) are shifted into the higher surface area per molecule in relation to the DODAB isotherm (line 1, Figure 4A,B). In this case, besides electrostatic interaction between the positively charged DODAB monolayer and the negatively charged DNA phosphates, the hydrophobic insertion of cholesterol moieties into the monolayer should be considered. The later process adds additional surface area to the DODAB film that is related to the cross-section of cholesterol moiety. The addition of potassium ions (line 6, Figure 4A,B) and sodium ions (line 7, Figure 4A,B) for cholesterol-anchors probes, induced G-quadruplex folding that resulted, similar to the probe without cholesterol (F-TBA-T, py-TBA-py), in a more compact monolayer formation compared to the monolayer for the probe in the absence of metal ions (line 5, Figure 4A,B). 

**Figure 5:**
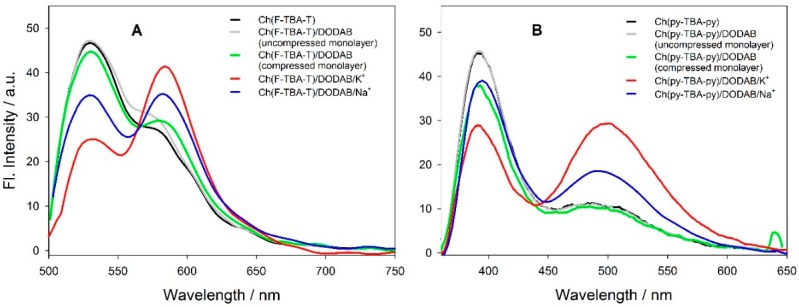
Emission spectra for Ch(F-TBA-T) (**A**) and Ch(py-TBA-py) (**B**) probes at monolayer DODAB interface: probe in subphase (black line), after DODAB monolayer spreading (grey line); after monolayer compression to 20 mN/m (green line); after K^+^ addition (red line); and, after Na^+^ addition (blue line). *Conditions:* subphase contained 10 mM TA buffer (pH = 7.5) and 40.0 nM probe; 100 mM KCl or NaCl.

Fluorescence spectra of probes were recorded with a bifurcated fiber optic accessory that was finely positioned above the monolayer surface at an angle of ca. 45° and coupled to the spectrofluorimeter (Figure S1, ESI). The excitation wavelength was set at 470 nm for the FRET-labeled probes and at 330 nm for the pyrene-modified probes in order to minimize scattering at the spectral range of interest. The background scattering spectrum of the DODAB monolayer on a buffer subphase was subtracted from each spectrum of oligonucleotide probe. G-quadruplex probes dynamically adsorbed or anchored at the monolayer interface are expected to respond to the presence and the concentration of metal cations such as Na^+^ and K^+^. Figure 5 shows fluorescence spectra of monolayer-anchored probes (Ch(F-TBA-T), Ch(py-TBA-py)) recorded for a relaxed and compressed monolayer, and after the addition of K^+^ or Na^+^ ions. Fluorescence spectra for monolayer-adsorbed probes (F-TBA-T, py-TBA-py) under the same conditions are shown in Figure S9, ESI. One can notice that the positions of fluorescence bands in the monolayer spectra are similar to those for probes in solution (Figure 1 and Figure S3, ESI), which indicates that adsorption or anchoring of probes at the monolayer interface imposes negligible effects on the spectral properties of fluorophores. Only in the case of pyrene-modified probes (py-TBA-py, Ch(py-TBA-py)), does the monomer band at 390 nm not show the vibronic structure that is probably connected with stacking interactions of the pyrene fluorophore with nucleobases. The presence of the DODAB monolayer (grey line in Figure 5) does not practically affect the emission spectra of probes recorded in solution. However, compression of this relaxed monolayer to 20 mN/m (green line), caused a slight increase in intensity of fluorescence bands. The addition of potassium (red line in Figure 5) and sodium cation (blue line in Figure 5) causes analogous changes, as in the case of experiments performed in solution. Specifically, the FAM fluorescence band (λ = 520 nm) is quenched, while TAMRA emission at 583 nm is enhanced due to FRET sensitization for Ch(F-TBA-T) (Figure 5A) and F-TBA-T (Figure S9B, ESI) probes. Similarly, the appearance of a new broad excimer band around 480 nm, accompanied by quenching of the monomer emission (380 nm) is observed for Ch(py-TBA-py) (Figure 5B) and py-TBA-py (Figure S9B, ESI) probes. As expected, all probes show more efficient spectral changes for potassium ions in accordance with significantly higher binding preferences of probes to K^+^ over Na^+^ ions. It should be noted that the addition of metal cations behind the barriers did not exert an immediate effect on the fluorescence characteristics of the probe. The lack of fast response of the probe to the introduced cations can be rationalized in terms of a diffusion process that cannot be accelerated by, for example, stirring. However, moving the barriers backwards and re-compressing the monolayer (with adsorbed or anchored probe molecules) diminished the problem with slow diffusion of metal cation and enabled folding of the probe into G-quadruplexes. The appropriate time of equilibration (~60 min.) enabled effective and reproducible monitoring of spectral changes caused by the presence of cations (K^+^, Na^+^).

### 3.4. Fluorescent Bioimaging of Probes in HeLa Cells

**Figure 6:**
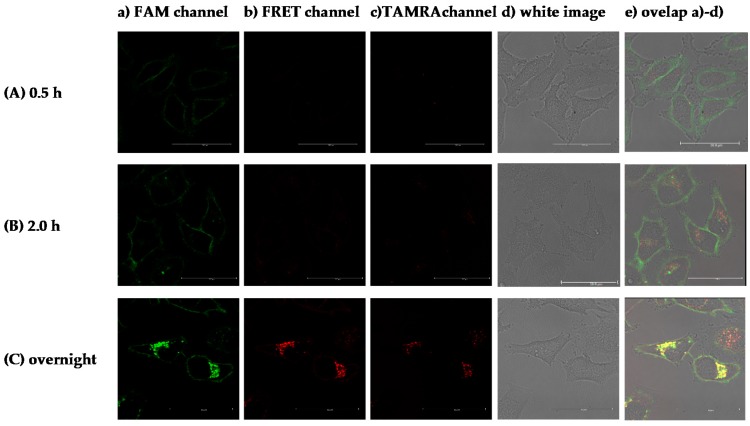
Confocal microscopy analysis of HeLa cells treated with 50 nM Ch(F-TBA-T) for 0.5 h (**A**), 2 h (**B**) and overnight (**C**). Fluorescence emission filters: (1) for FAM channel (green), 510–540 nm, excitation wavelength: 480 nm; (2) for TAMRA channel (red), 595–630 nm, excitation wavelength: 560 nm; and, (3) for FRET channel, 595–630 nm, excitation wavelength: 480 nm. Scale bars: 50 μm.

To visualize the successful anchoring of the Ch(F-TBA-T) to the outer cell membrane, we imaged the probes on the cell membrane of HeLa cells using confocal fluorescence microscopy. As shown in Figure 6A, after incubation for 0.5 h, most of the probes are located on the cell membrane. The internalization of the Ch(F-TBA-T) probes was investigated by incubating cells with the probe at 37 °C for different time intervals. Fluorescence images taken after 2 h (Figure 6B), 3.5 h (Figure S10, ESI) and 24 h (Figure 6C) of incubation indicated that a significant fraction of the probe was still present on the membrane of the cells; however, some of the probe molecules were clearly seen inside the cells. 

**Figure 7:**
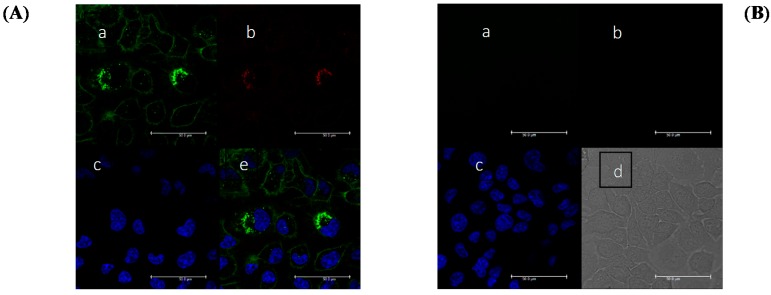
Confocal microscopy analysis of HeLa cells treated with 200 nM Ch(F-TBA-T) (panel (**A**)) and untreated (negative control in panel (**B**)): (a) FAM channel (green); (b) FRET channel; (c) Hoechst 33342 fluorescence form stained nuclei (blue); as well as (d) bright field image of control and (e) overlay of all images a–c of Ch(F-TBA-T). The experiment details: The probe was added at 200 nM concentration to medium. After leaving overnight, Hoechest 33342 was applied to cell culture at a final concentration of 5 µg/mL and images was were taken after 15 min. of staining. Before fluorescence imaging, experiment cells were washed twice with PBS and placed in FluoroBright Live Cell Fluorescence Imaging Medium. Fluorescence emission filters: (1) for FAM channel, 510–540 nm, excitation wavelength: 480 nm; (2) for Hoechst channel, 480–500 nm, excitation wavelength: 405 nm;and (3), for FRET channel, 595–630 nm, excitation wavelength: 480 nm. Scale bars: 50 μm.

In order to determine whether the internalized Ch(F-TBA-T) molecules are accumulated in the nucleus, we performed an additional experiment with Hoechst 33342. Hoechst is a popular cell-permeant nuclear counterstain that emits blue fluorescence when bound to dsDNA (Figure 7B) [48]. Therefore, we added Hoechst into cells loaded previously with Ch(F-TBA-T). Co-localization images between Ch(F-TBA-T) probes and Hoechst clearly indicate that Ch(F-TBA-T) are not located in the nucleus (Figure 7A). Moreover, the ability of Ch(F-TBA-T) to enter the cells can be attributed to the presence of cholesterol moiety as the analogous probe without cholesterol moiety (F-TBA-T) was not only unable to anchor to the cell membrane, but also did not penetrate inside the cell (compare Figure 7A with Figure S10B, ESI). In the case of Ch(py-TBA-py), the situation seems to be a little more complicated as we were not able to observe fluorescence signal from the membrane bound probe but only from the probe located inside the cells (Figure S11, ESI). These results suggest the Ch(py-TBA-py) probe was able to cross the membrane due to presence of the cholesterol moiety. On the other hand, the Ch(py-TBA-py) probe has been previously shown to anchor into the DODAB monolayer; thus, a lack of fluorescence signal from the cell membrane bound probe may indicate both quenching by membrane constituents (e.g., proteins) or high membrane permeability of the probe enhanced by the presence of hydrophobic pyrene tags. For further evaluation of the potential applications of the Ch(F-TBA-T) as a potassium ion indicator, we are planning to perform experiments in cellulo with drugs able to change the potassium level.

## 4. Conclusions

Two cholesterol-bearing fluorescent potassium probes (Ch(F-TBA-T), Ch(py-TBA-py)) based on G-quadruplexes, for spontaneous anchoring into the hydrophobic interior of lipid structures, were designed. The oligonucleotide probes preserved their abilities to fold into a G-quadruplex structure and to interact with metal cations with binding affinity and selectivity comparable to unmodified oligonucleotides. All probes exhibited higher potassium ion binding affinity and advantageous K^+^/Na^+^ selectivity.

We also reported the G-quadruplex folding ability of cholesterol-anchored fluorescent oligonucleotide probes at the Langmuir monolayer interface, which mimicked the biomembrane surface. The monolayer (cell surface) interaction with the probe is dictated by the physicochemical properties of the probe and the monolayer (cell) interface. In our study, there were two major driving forces involved in the monolayer/probe interaction: (i) the hydrophobic insertion of cholesterol moieties into the membrane; and (ii), the electrostatic interaction between the positively charged DODAB monolayer and the negatively charged DNA. The fluorescent probes without cholesteryl-TEG anchors (F-TBA-T, py-TBA-py) appeared to bind membranes with positive charge (i.e., DODAB), which is expected for the negatively charged polyelectrolytes like DNA. On the other hand, the cholesterol-anchored probes (Ch(F-TBA-T), Ch(py-TBA-py)) penetrated the hydrophobic domain of the lipid monolayer. Based on confocal fluorescence microscopy, the cholesterol-containing fluorescent oligonucleotide probes (Ch(F-TBA-T), Ch(py-TBA-py)) were shown to be located on the cell membrane of HeLa cells.

## Figures and Tables

**Table 1:** Fluorescent oligonucleotide probes used in this study.

Abbreviation of Probes	Sequence
F-TBA-T	5′-FAM-GGT TGG TGT GGT TGG ATT T-TAMRA-3′
Ch(F-TBA-T)	5-Ch-(dT FAM)-TTT AGG TTG GTG TGG TTG GAT TT-TAMRA-3′
py-TBA-py	5′-py-GGT TGG TGT GGT TGG-py-3′
Ch(py-TBA-py)	5′-Ch-py-TGG TTG GTG TGG TTG G-py-3′
TBA	5′-TGG TTG GTG TGG TT GG-3′

**Table 2:** Melting temperatures (Tm) for quadruplex-forming oligonucleotide probes determined from dependences of absorbance at 295 nm in the presence K^+^ and Na^+^ ions.

Abbreviation of Probes	Tm/K^+^ (°C)	Tm/Na^+^ (°C)
F-TBA-T	38.0	23.0
Ch(F-TBA-T)	48.0	39.0
py-TBA-py	55.0	25.0
Ch(py-TBA-py)	77.0	43.0
TBA	53.0	24.0

## Supplementary Materials

Supplementary materials are available online at http://www.mdpi.com/1424-8220/18/7/2201/s1.
