# Supplementary material for: Cholesterol-Bearing Fluorescent G-Quadruplex Potassium Probes for Anchoring at the Langmuir Monolayer and Cell Membrane
Source: Sensors (Basel). 2018 Jul 9;18(7):2201. doi: 10.3390/s18072201 (PMC6069133; doi:10.3390/s18072201)
Supplement: Supplementary file 1 [file sensors-18-02201-s001.pdf]

Electronic Supplementary Information

Article

Cholesterol-Bearing Fluorescent G-Quadruplex Potassium Probes for Anchoring at the Langmuir Monolayer and Cell Membrane

Angelika Świtalska [1,*], Anna Dembska [1], Agnieszka Fedoruk-Wyszomirska [2] and Bernard Juskowiak [1]

1 Faculty of Chemistry, Adam Mickiewicz University, Umultowska 89b, 61-614 Poznan, Poland; aniojka@amu.edu.pl (A.D.); juskowia@amu.edu.pl (B.J.)

2 Institute of Bioorganic Chemistry, Polish Academy of Sciences, Noskowskiego 12/14, 60-704 Poznan, Poland; agaw@ibch.poznan.pl

* Correspondence: switalskaang@gmail.com or angelika.swiatkowska@amu.edu.pl; Tel.: +48-618-291-771

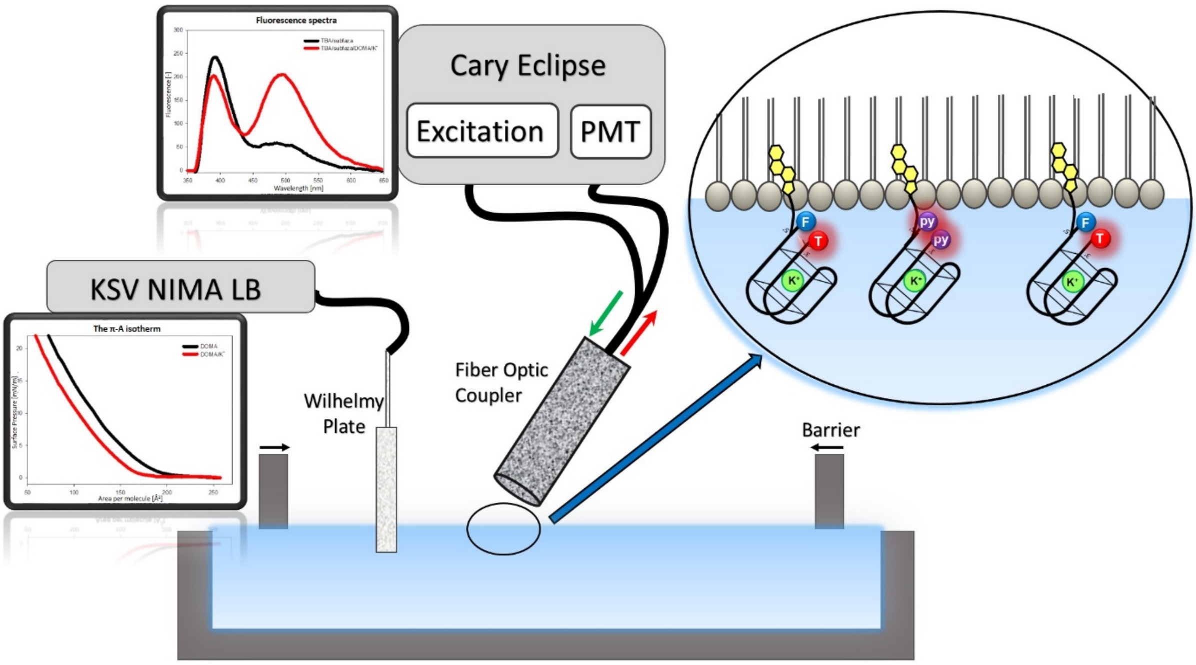

Scheme S1. Experimental setup for fluorescence measurements of monolayer adsorbed probes, consisting of Langmuir trough and spectrofluorimeter equipped with a fiber optic accessory.

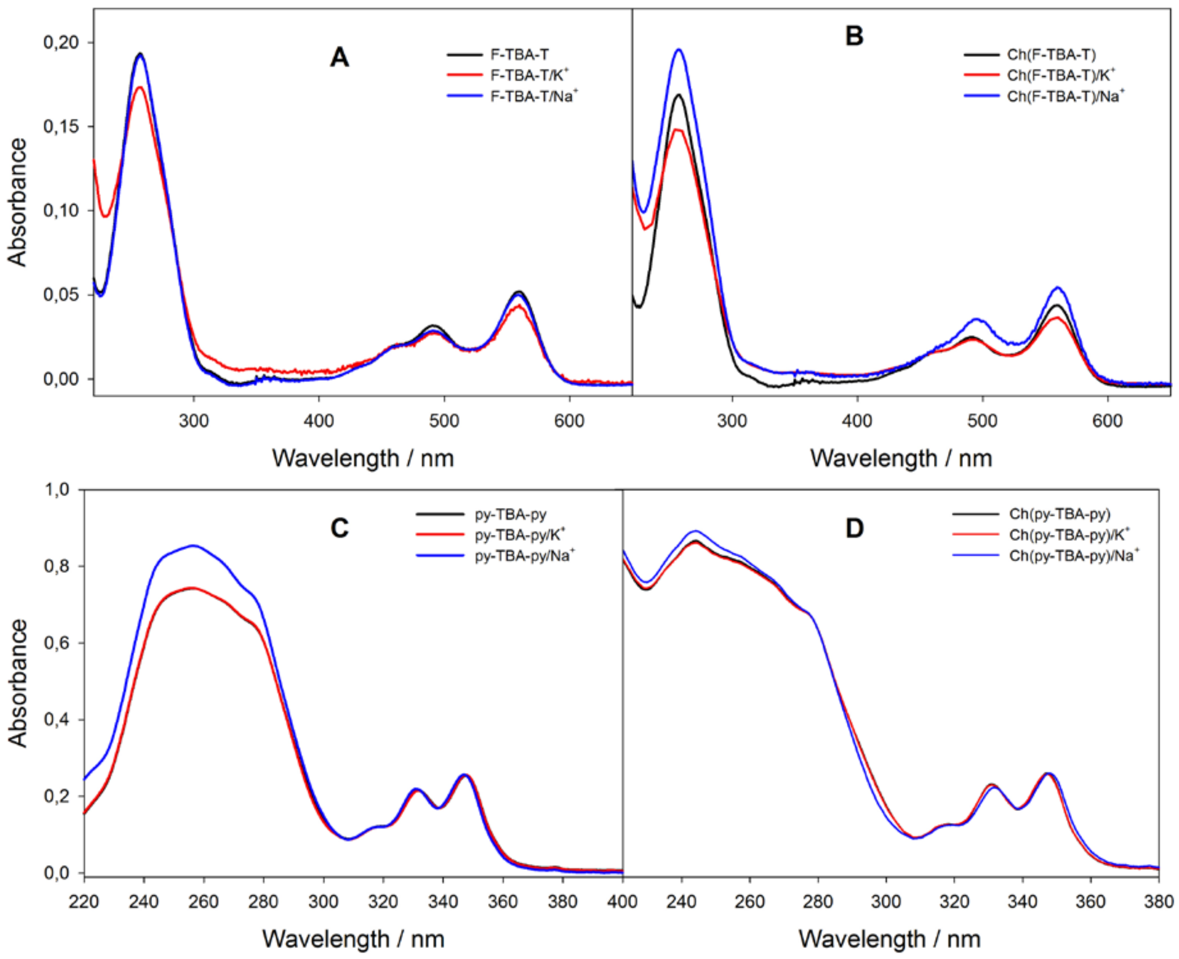

Figure S1. Absorption spectra of FRET-labeled (**A, B**) and pyrene-labeled probes (**C, D**). *Conditions*: 10 mM TEA buffer (pH 7.5).

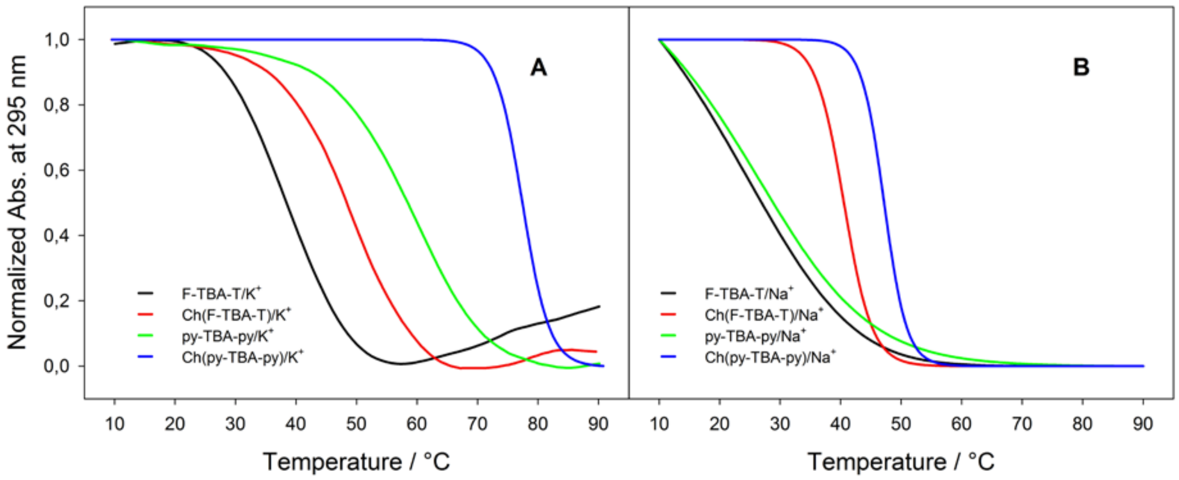

Figure S2. Normalized UV melting curves of 1 μM F-TBA-T (black line), Ch(F-TBA-T) (red line), py-TBA-py (green line) and Ch(py-TBA-py) (blue line) in the presence K^+ (**A**) and Na^+ (**B**), respectively, at 295 nm. *Conditions*: 100 mM KCl or NaCl, 10 mM TEA buffer (pH 7.5).

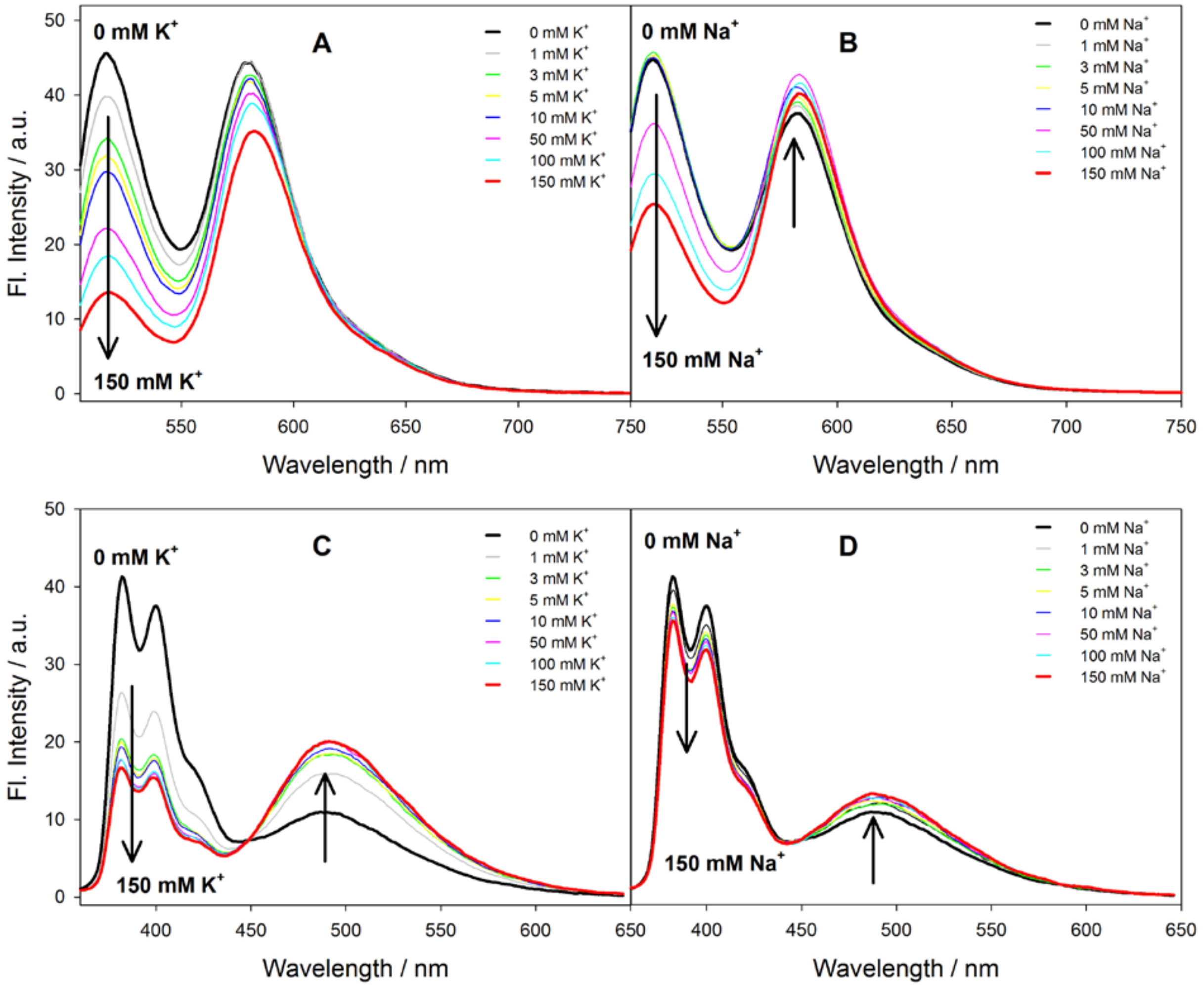

Figure S3. Potassium and sodium ions effect on the fluorescence spectra of 0.2 μM F-TBA-T (**A**, **B**, respectively) with λ_{ex} = 490 nm and py-TBA-py probe (**C**, **D**, respectively) with λ_{ex} = 340 nm. *Conditions*: 10 mM TEA buffer (pH 7.5), KCl and NaCl conc: 1, 3, 5, 10, 50, 100, 150 mM.

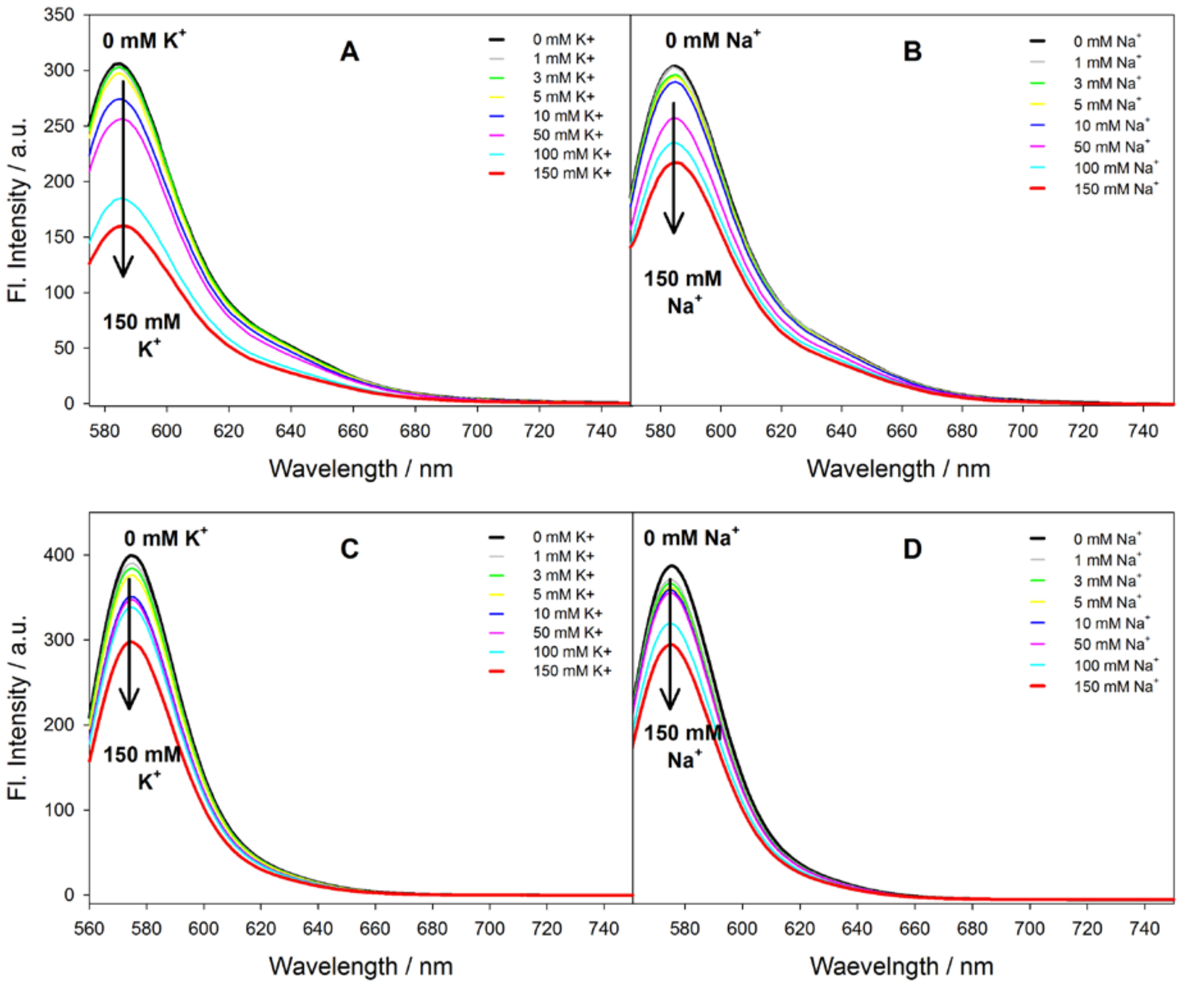

Figure S4. Potassium and sodium ions effect on the fluorescence spectra of 0.2 μM F-TBA-T (**A**, **B**, respectively) and Ch(F-TBA-T) (**C**, **D**, respectively) probes with λ_{ex} = 560 nm. *Conditions*: 10 mM TEA buffer (pH 7.5), KCl and NaCl conc: 1, 3, 5, 10, 50, 100, 150 mM.

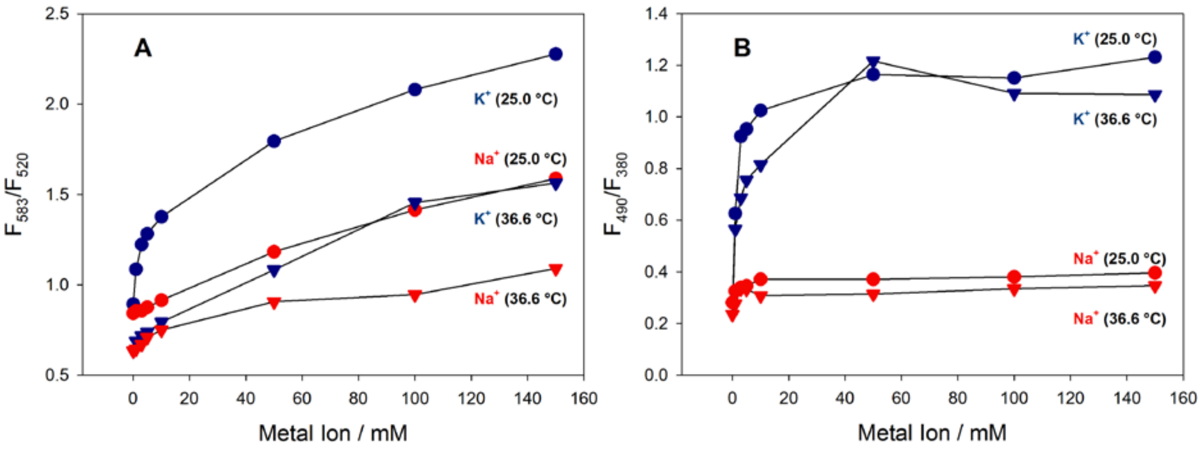

Figure S5. Fluorescence intensity ratio (F_{583}/F_{520}) for F-TBA-T (**A**) and F_{490}/F_{380} ratio for py-TBA-py (**B**) plotted against K^+ or Na^+ concentration at 25 °C and 36.6 °C.

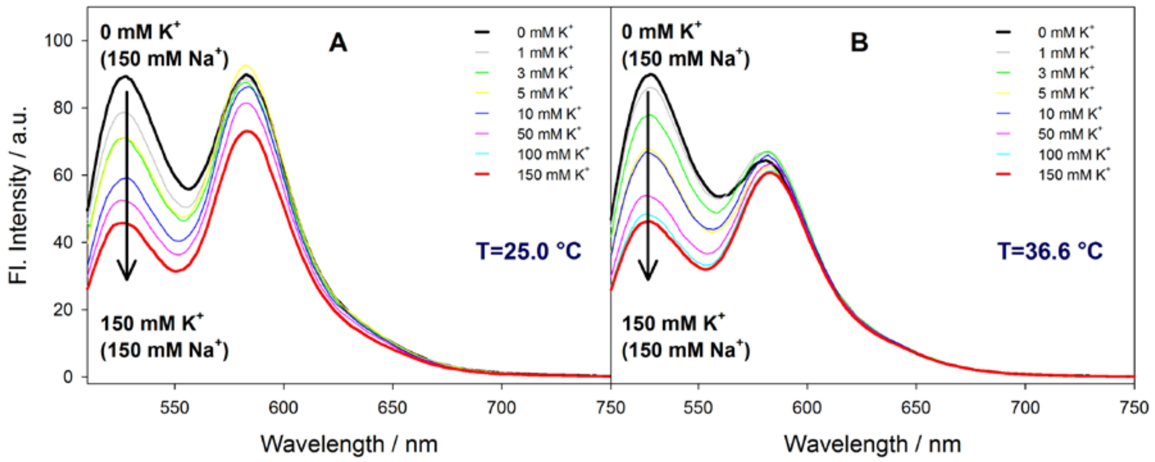

Figure S6. Potassium ions effect in the presence of 150 mM Na^+ ion on the fluorescence spectra of 0.2 μM Ch(F-TBA-T) at 25.0 °C (**A**) and 36.6 °C (**B**) with λ_{ex} = 490 nm. *Conditions*: 10 mM TEA buffer (pH 7.5), 150 mM NaCl, KCl conc: 1, 3, 5, 10, 50, 100, 150 mM.

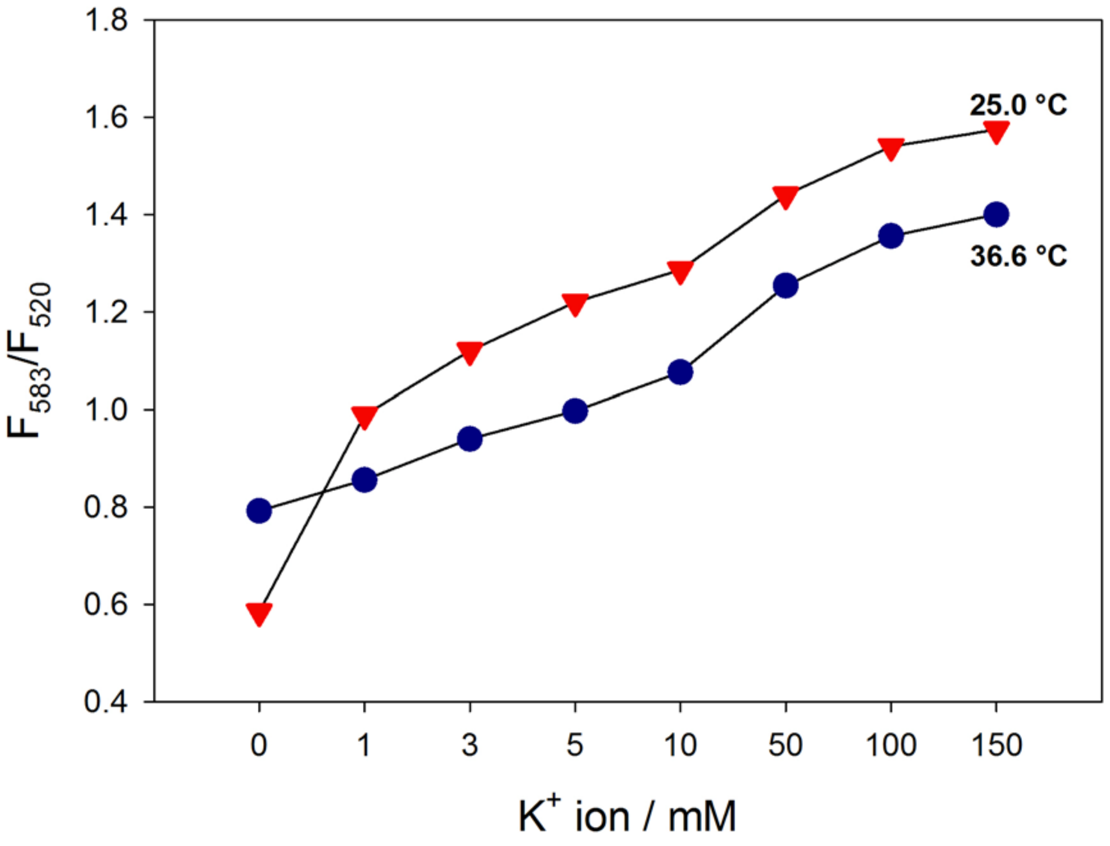

Figure S7. Fluorescence intensity ratio (F_{583}/F_{520}) for Ch(F-TBA-T) (**A**) plotted against K^+ concentration in the presence 150 mM Na^+ at 25 °C (triangles) and 36.6 °C (circles).

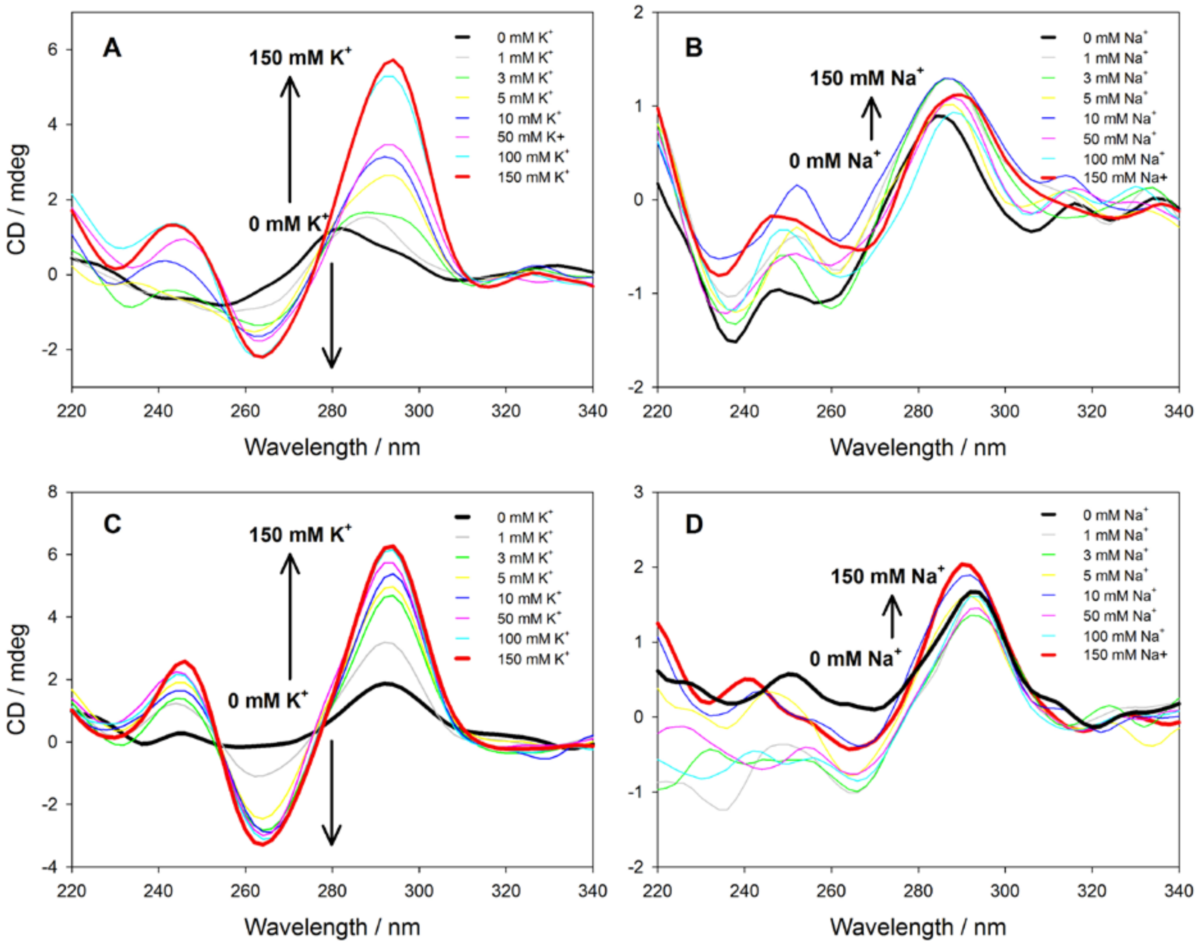

Figure S8. Potassium and sodium ions effect on the CD spectra of 1 μM F-TBA-T (**A**,**B**,) and py-TBA-py probe (**C**, **D**). *Conditions*: 10 mM TEA buffer (pH 7.5), KCl and NaCl conc: 1, 3, 5, 10, 50, 100, 150 mM.

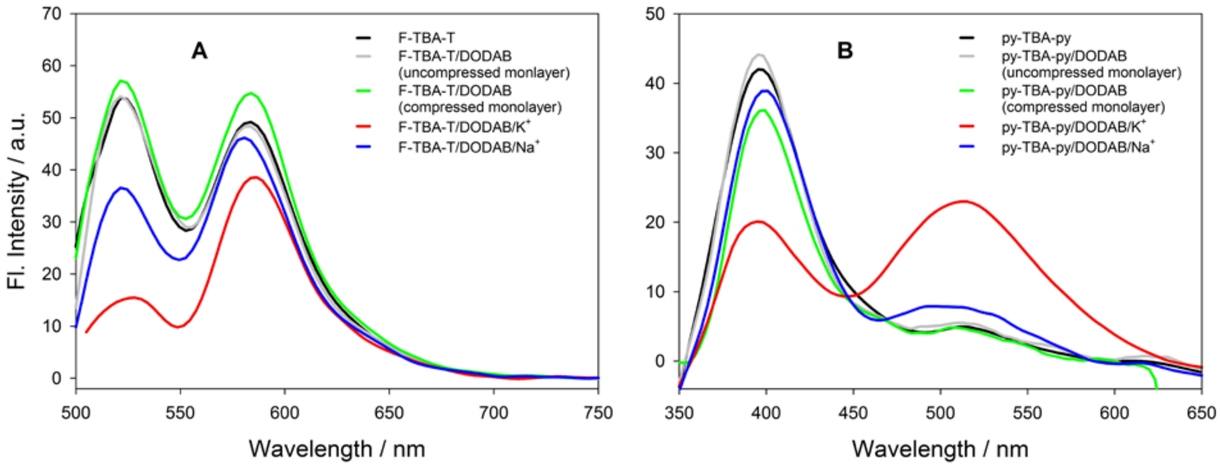

Figure S9. Emission spectra for F-TBA-T (**A**) and py-TBA-py (**B**) probes at monolayer DODAB interface: probe in subphase (black line), after DODAB monolayer spreading (grey line), after monolayer compression to 20 mN/m (green line), after K^+ addition (red line), and after Na^+ addition (blue line). Conditions: subphase contained 10 mM TA buffer (pH = 7.5) and 8.3 nM probe; 100 mM KCl or NaCl.

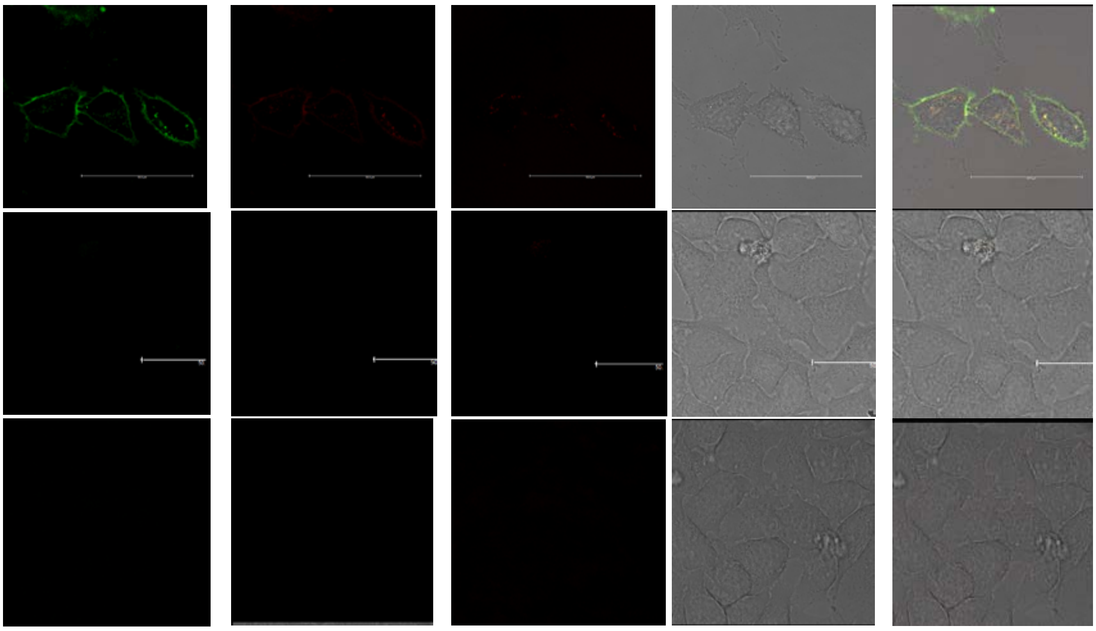

Figure S10. Fluorescence images of HeLa cells treated with 50 nM Ch(F-TBA-T) for 3.5 h (panel A), with 50 nM F-TBA-T overnight (panel B) as well as control containing untreated cells (panel C): a) FAM channel, green; b) FRET channel, red; c) TAMRA channel, red; and d) bright field image and e) overlay of all images. Fluorescence emission filters: 1) FAM channel, 510-540 nm; excitation wavelength: 480 nm; 2) TAMRA channel, 595-630 nm; excitation wavelength: 560 nm and 3) FRET channel, 595-630 nm; excitation wavelength: 480 nm. Scale bars: 50 µm.

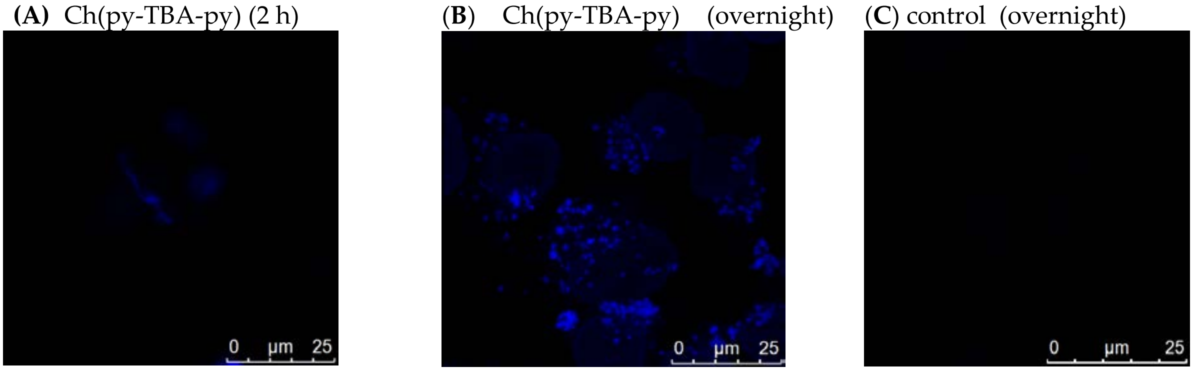

Figure S11. Fluorescence images of HeLa cells treated with 200 nM Ch(py-TBA-py) for 2 h (panel A), overnight (panel B) as well as control containing untreated cells (panel C). Fluorescence emission filter: 480-500 nm; excitation wavelength: 405 nm. Scale bars: 25 µm.
